# Exosomal RNAs in the development and treatment of pituitary adenomas

**DOI:** 10.3389/fendo.2023.1142494

**Published:** 2023-02-17

**Authors:** Mengqi Chang, Shenzhong Jiang, Xiaopeng Guo, Jun Gao, Peng Liu, Xinjie Bao, Ming Feng, Renzhi Wang

**Affiliations:** ^1^ Department of Neurosurgery, China Pituitary Disease Registry Center, Peking Union Medical College Hospital, Peking Union Medical College & Chinese Academy of Medical Sciences, Beijing, China; ^2^ Medical Research Center, Peking Union Medical College Hospital, Chinese Academy of Medical Sciences, Beijing, China

**Keywords:** pituitary adenoma, exosome, biomarker, noncoding RNA, treatment therapy

## Abstract

Exosomes are small extracellular vesicles that carry various bioactive molecules including various RNAs that modulate the activities of recipient cells. It has drawn considerable attention as means of cell communication and drug delivery. Exosome plays important role in various tumors, but it is rarely summarized in pituitary adenoma (PA). PA is the second most common primary central nervous system tumor, and its recurrence and persistent postoperative hormone hypersecretion lead to compromised quality of life. How exactly exosomes impact tumor development and hormone secretion is important for the development of this tumor diagnosis and treatment. In this review, we discuss how exosomal RNAs impact PAs and their potential as future clinical therapies. In our literature review, first, we found that exosomal microRNA hsa-miR-1180-3p is a potential early biomarker for NFPAs. Since NFPAs are typically difficult to diagnose, this is an especially important finding. Second, exosomal protein transcripts are potential invasive biomarker, such as *MMP1*, *N-cadherin*, *CDK6*, *RHOU*, *INSM1*, and *RASSF10*. Third, exosomal contents such as hsa-miR-21-5p promote distant bone formation of GHPA patients. Fourth, tumor suppressors in the exosome constitute novel therapeutic application of exosome, including long noncoding RNA (lncRNA) H19, miR-149-5p, miR-99a-3p, and miR-423-5p. This review discusses the possible mechanisms of exosome and their contents in PA and promotes the use of exosomes in both clinical diagnosis and treatment of this tumor.

## Introduction

**Figure 1:**
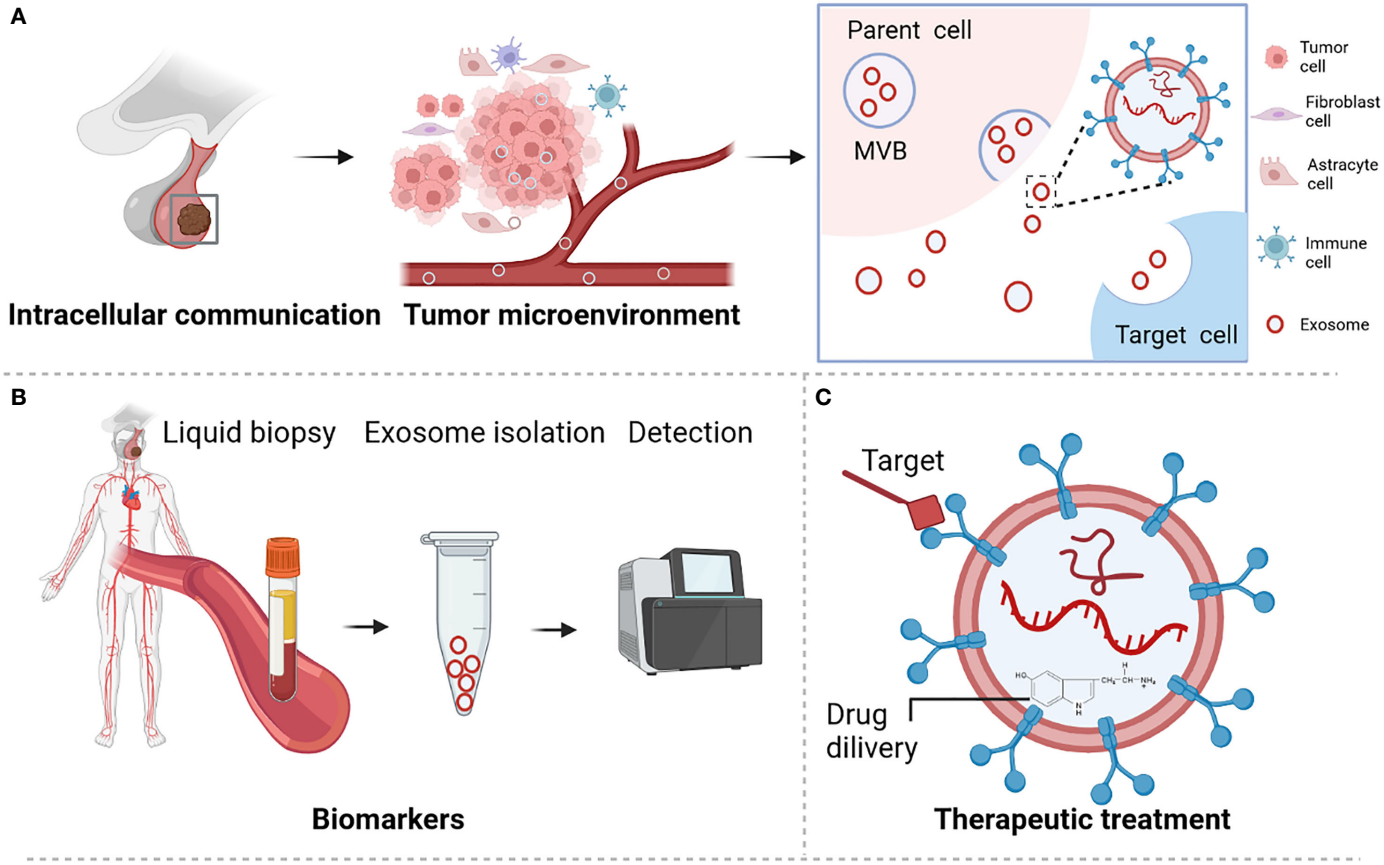
Role and functions of exosome in pituitary adenoma. **(A)** Exosome participates in microenvironment regulation and cell communication. **(B)** Exosome served as biomarker for PA disease. **(C)** Exosome is a promising novel treatment therapy. Figures are created in BioRender.com.

Pituitary adenoma (PA) is a common type of brain tumor that accounts for approximately 17.2% of intracranial tumors (1, 2). These tumors are classified according to the hormone they excessively secrete. Accordingly, they include prolactin-secreting pituitary adenoma (PRLPA), non-secreting pituitary adenoma (NFPA), growth hormone-secreting pituitary adenoma (GHPA), as well as adrenocorticotropic hormone-secreting pituitary adenoma (ACTHPA) and other types of PA. Treatments for PAs include surgery, radiotherapy, somatostatin analogues, growth hormone antagonists or dopamine agonist (3–5). However, these treatments are challenging due to development of drug resistance, lack of treatment targets, and limited in-depth understanding of the molecular mechanisms responsible for the development of these tumors (3, 5). Various molecules are secreted by cells *via* different types of extracellular vesicles, including microvesicles, apoptotic bodies, and exosomes. Recently, exosomes have drawn considerable attention as a means of intercellular communication because they carry bioactive molecules that can modulate the activities of recipient cells (6, 7). Additionally, exosomes are promising biomarkers and vehicles for drug delivery (**Figure 1**) (8, 9). Exosomes are small extracellular vesicles with average diameters of 40–150 nm. They are secreted by a wide variety of cells and have been detected in nearly all body fluids, including blood, urine, cerebrospinal fluid, and saliva (8, 9). The secretory quantity and content of exosomes can vary according to their biogenesis, cell of origin, and physiological or pathological cell status. Among these, noncoding RNAs (ncRNAs) are enriched and stable in exosomes and are important owing to their regulatory function in tumor initiation and progression, including in PA (10, 11).

### Biogenesis and characteristics of exosomes

Exosome biogenesis begins at the cell membrane and is connected to endosomal uptake and form the early endosomes. Then, multivesicular bodies (MVBs) formation through inward budding of early endosomes and selected contents including RNAs, proteins or lipid. Finally, the MVB was fused with cell membrane leading to the release of exosome, or fuse with lysosome result in the degradation of exosome. During this process, MVBs are generated through two distinct mechanisms. One pathway involves a large multi-subunit protein complex called the endosomal sorting complex required for transport (ESCRT) mechanism, whereas the second pathway is an ESCRT-independent mechanism. ESCRT is an important mechanism of MVB synthesis as it guides the packaging of molecules within the exosomes of MVBs (12). Usually, MVBs form in an ESCRT-independent manner (13). However, the mechanism of ESCRT-independent exosome formation and its regulation in PA remain largely unknown.

Given their cell-specific uptake by extracellular receptors and their ubiquity in cell signaling, exosomes are suitable candidates as disease biomarkers and drug delivery vehicles (8). Next, we discuss the types of molecules identified in exosomes and their potential roles in PAs. These molecules include proteins, lipids, and nucleic acids, among which ncRNAs are more abundant in exosomes (14). In this review, we summarized the current understanding of the role of exosomal RNA and more specifically of ncRNAs, in relation to PA progression and its potential clinical applications.

### Background of noncoding RNA in PAs

**Figure 2:**
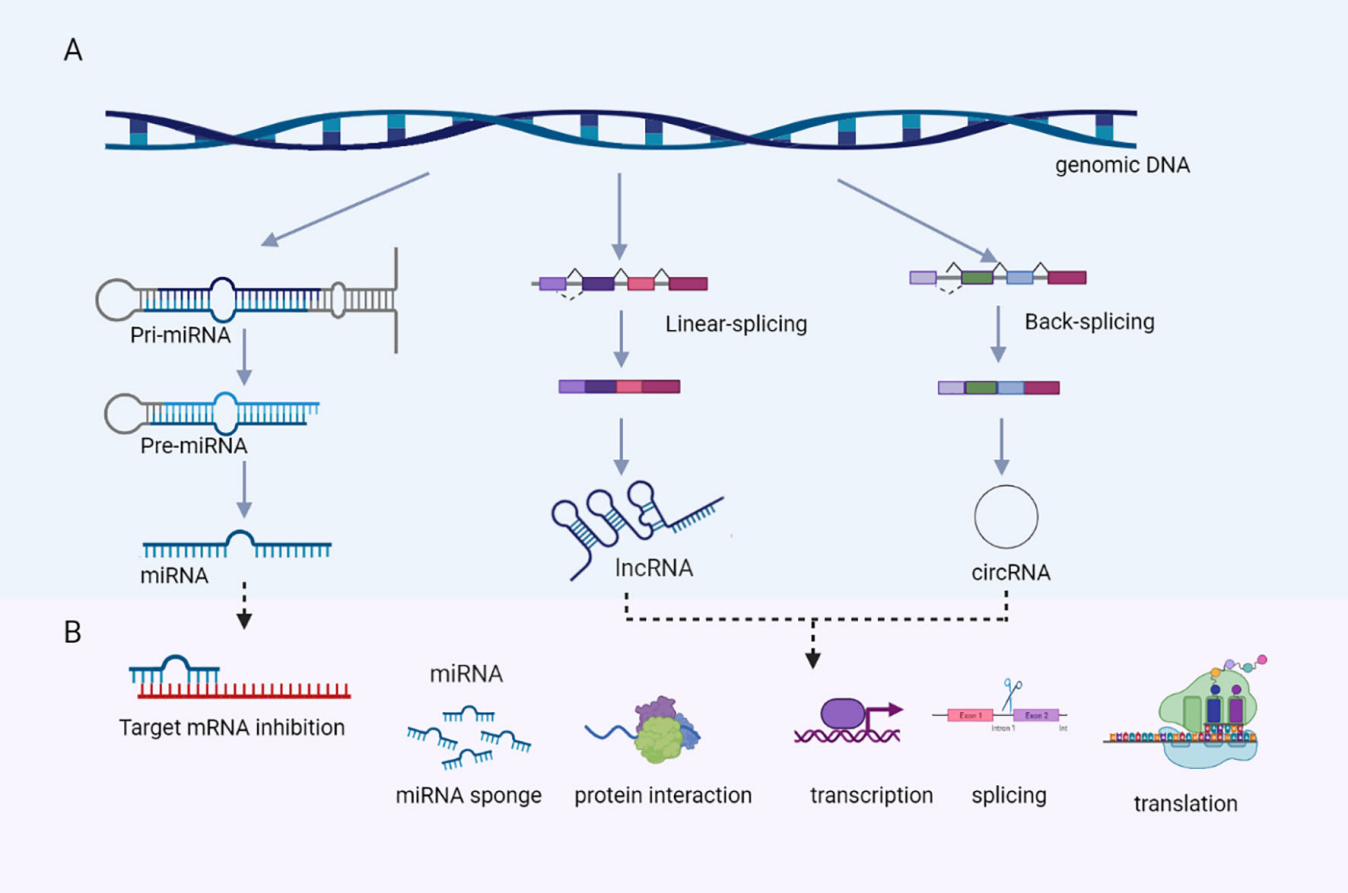
Genesis **(A)** and biological functions **(B)** of ncRNAs, including miRNA, lncRNA, and circRNA. Figures are created in BioRender.com.

Although mRNAs were reported to play important role in tumor genesis, tumor development and treatment of PAs, ncRNAs which are enriched in exosomes have drawn more attention than mRNAs, because ncRNAs are usually much shorter and are easier to be contained in exosomes. Exosomes usually contain different types of ncRNAs, including microRNAs (miRNAs), long noncoding RNAs (lncRNAs), circular RNAs (circRNAs), and other ncRNAs. Different types of ncRNAs were all reported to be involved in tumor disorders (15, 16) (**Figure 2**).

### MicroRNA in PAs

miRNAs are small ncRNA molecules that consist of approximately 21–25 nucleotides (17). The pri-miRNA is cleaved into precursor miRNA (called pre-miRNA) which is an 85-nucleotide stem-loop structure by a complex containing DGCR8 protein. Then Dicer processes hairpin structured precursor miRNA into mature miRNA (18). These mature miRNAs repress gene expression by binding to 3′ untranslated regions of target mRNAs, through which they impair mRNA stability and/or inhibit mRNA translation (17).

In PAs, miRNA expression is associated with PA tumorigenesis and treatment resistance. For example, AIP expression is negatively correlated with miR-34a in GHPAs, where elevated miR-34a levels promote the resistance to octreotide (19–22). Differentially expressed miRNAs have recently been discovered identified in exosomes of PA patients, suggesting that miRNAs are selectively sorted into exosomes participating in tumor progression, invasion, and non-hormonal action. Thus, these miRNAs represent potential markers for the diagnosis or treatment of PAs (23).

### Long noncoding RNA in PAs

LncRNA is noncoding RNAs that are longer than 200 nucleotides (24). LncRNA modulates gene expression through multiple mechanisms: (1) precursor of small RNAs such as miRNA (2) interaction with regulatory protein. (3) cis and/or in trans regulation of transcription (4) regulation of mRNA alternative splicing (5) lncRNAs can occasionally encode small peptides (25).

LncRNAs in PAs involves in hormone secretion (26), tumor suppress (27), tumor progress (28), recurrence prediction (29), as well as tumor invasion (30, 31). Furthermore, dysregulation of lncRNA expression has been observed in exosomes of bone-invading PAs. Hence, exosomal lncRNAs should be explored as biomarkers for both diagnosis and prognosis (32).

### Circular RNA in PAs

Circular RNA (circRNA) owes its name to its closed-loop structure. CircRNAs were produced from corresponding pre-mRNAs *via* back-splicing (33),. These circRNAs regulate genes *via* two pathways: (1) by acting as a miRNA “sponges”, thereby affecting miRNA target genes; (2) by binding to specific RNA-binding proteins and impacting their functions (34, 35). A small part of circRNAs also could encode small peptides.

Interestingly, circRNAs are more abundant in the brain compared to other organs, thereby indicating that circRNA is important for brain function and brain diseases (36). Furthermore, many studies have shown that circRNAs are aberrantly expressed in PAs and are linked to proliferation (37), progression (38), invasion (39) and GH secretion in pituitary somatotroph adenoma (40). Several studies have identified circRNA in exosomes (41, 42). Notably, these studies showed that these circRNAs initiate their circulation process inside exosomes before reaching their respective recipient cells, where they carry out various biological functions (41, 43). Unlike linear RNAs, circRNAs are more suitable as biomarkers because their loop structures remain stable for a longer half-life (44, 45). However, the role of exosomal circRNA in PA is largely unknown. Therefore, investigation of exosomal circRNA in PA is a potential direction for PA diagnosis and treatment therapy.

## Potential clinical applications of exosomal RNA in pituitary adenoma

Since there is a part of PAs exhibit increased cell proliferation, invasive propensities, excess secretion of various hormones, and even drug resistance. However, the mechanisms that underlie these behaviors remain unclear. Increasing evidence suggests that exosomes increase pituitary tumor progression *via* transfer of bioactive molecules between different cell populations (24, 46). As important exosomal components, RNAs, especially ncRNAs play key roles in multiple aspects of tumor biology, including proliferation, migration, and tumor microenvironment (10). In the following sections, we summarize the current understanding of the specific roles and mechanisms of exosomal RNAs in the progression of PAs (**Table 1**).

**Table 1:** Biological functions of exosomal RNAs in PA.

Candidates	Subtype	Function	Reference
hsa-miR-486-5p	NF-PA	Early diagnosis	47
MMP1	Invasive NF-PA	Invasive tumor diagnosis	40
N-cad	Invasive PA	Invasive tumor diagnosis	48
CDK6 and RHOU	Invasive NF-PA	Invasive tumor diagnosis	49
INSM1	Invasive NF-PA	Invasive tumor diagnosis	50
RASSF10	Invasive GH-PA	Tumor invasion	51
LncRNA H19	PRL-PA	Tumor suppressor	52
miR-149-5p /miR-99a	Invasive PA	Tumor suppressor	46
miR-7a-2-3p/miR-129-5p/miR-141-3p/miR-183-5p/miR183-5p/miR-200a-3p/miR-200b-5p/miR-204-5p/miR-340-5p/miR-375-3p/miR-497-5p	GH-PA	Tumor suppressor	11
miR-423-5p	GH-PA	Tumor suppressor	53
hsa-miR-21-5p	GH-PA	Osteoblast proliferation	54

### Exosomal biomarkers of NFPAs

#### Early diagnosis for NFPAs

**Figure 3:**
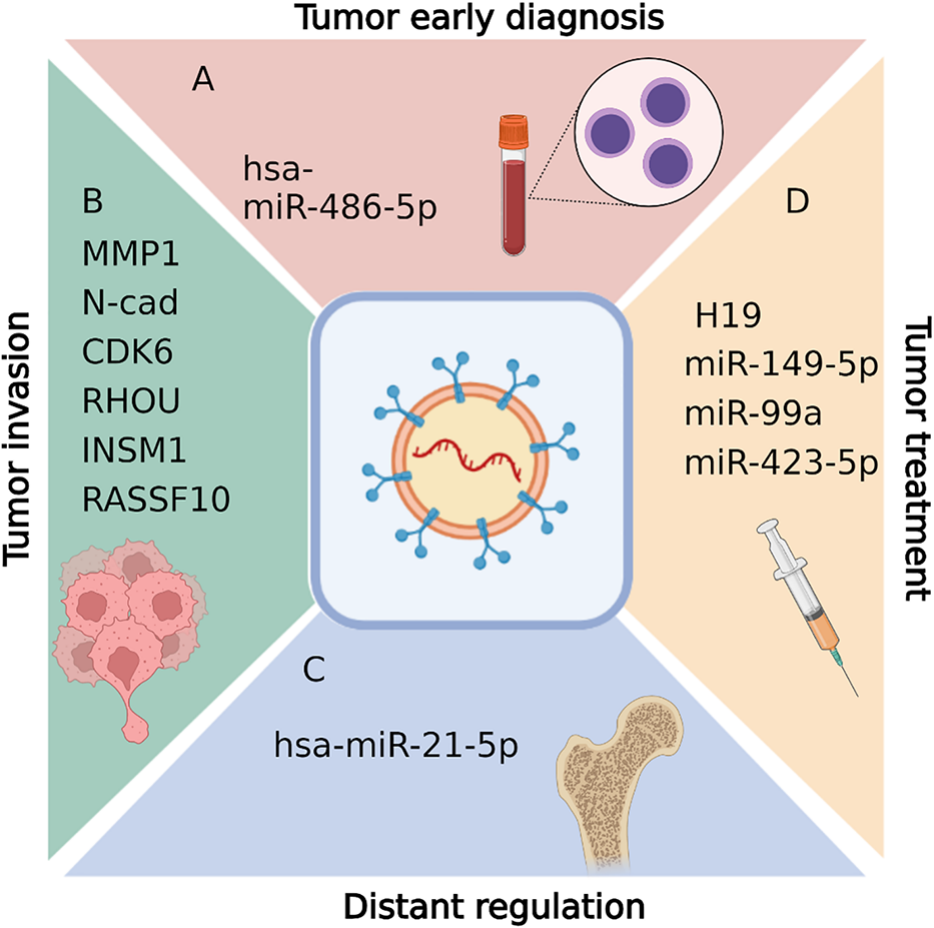
Biological functions of exosomal RNAs in pituitary adenomas, including tumor early diagnosis **(A)**, tumor invasion diagnosis **(B)**,distant regulation **(C)**, and tumor treatment **(D)**. Figures are created in BioRender.com.

Exosome is assumed to be a novel biomarker of NFPA for early diagnosis (**Figure 3A**). NFPA is one of the most common pituitary adenomas. Critically, complete resection has shown to be particularly difficult for larger and more aggressive tumors. Hence, early diagnosis is key to prevent recurrence. However, the lack of reliable screening approach for NFPAs is an urgent problem to be solved. To screen for NFPA and make prognostic predictions, Lyu et al. developed a method based on serum exosomal miRNA profiling (47). In that study, hsa-miR-486-5p, hsa-miR-151a-5p, hsa-miR-652-3p_R+1, and hsa-miR-1180-3p were identified as promising biomarkers for NFPA. Of those miRNAs, miR-486-5p is assumed to be the most accurate as an efficient biomarker for the prediction of progression and relapse among NFPA patients. Notably, this miRNA relates to tumor progression by epigenetically regulating MAPK signaling pathways (47).

#### Diagnosis for tumor invasion in NFPAs

Exosome also act as biomarkers in invasive PAs (**Figure 3B**). Tumor invasion into adjacent tissue is the main reason for PA recurrence, as invasion makes complete resection during surgery incredibly challenging. Thus, it is vital to understand tumor aggressive mechanisms and identify treatments that reduce invasion and improve PA surgical remission rates.

Several studies have identified different transcripts in exosomes as biomarkers for invasive PAs. It was reported that matrix metalloproteinase-1 (*MMP1*) (55), N-cadherin (48), E-cadherin (48), Epcam (48), CDK6 (49), RHOU (49), INSM1 (50) and RASSF10 (51) within the serum exosomes are elevated in invasive NFPA patients compared with noninvasive NFPA patients. Those disordered RNAs from exosome are potential diagnostic biomarkers for invasive PAs. In addition, the underlying regulation mechanisms are involved in cell migration (55), angiogenesis (55), epithelial-mesenchymal transition (48), and tumor microenvironment regulations (51). For example, enrichment of *MMP1* regulates the protease-activated receptor-1 signaling pathway, stimulating tumor migration, growth, and angiogenesis in PAs (55). Reduced expression of *RASSF10* can promote *MDM2* expression, impacting exosome secretion and therefore regulating the tumor microenvironment (51).

Together, those findings support the predictive value of *MMP1*, *INSM1*, *CDK6*, *RHOU*, *RASSF10*, and EMT-related marker mRNA expression in exosomes as *in situ* or circulating biomarkers for patients with invasive NFPA.

### Exosomal ncRNAs in disease regulation of hormone-secreting PA

#### Distant regulations caused by GHPAs

GHPAs usually result in disorders of organs distant from pituitary, which lead to clinical symptoms such as overgrowth of tissue, bone and multiple organs. Here we call it distant regulation of GHPA. In traditional concept, GH and insulin-like growth factor-1 (IGF-1) overproduction is the only reason for the distant disorders of GHPA patients. However, recently exosome was also indicated to exhibit distant regulations in GHPA disease progress (**Figure 3C**). Hyperostosis is one of the most common manifestations of acromegaly, which results from GH and insulin-like growth factor-1 (IGF-1) overproduction. However, current therapies focusing on the GH/IGF-1 axis are only effective in approximately 28%-50% of patients with acromegaly, while the remaining 50%-72% continue to experience excessive bone growth and/or abnormal bone metabolism, even after GHPA reduction or GH/IGF-1 level normalization (56–59).

Xiong et al. found that *in vitro*, GHPA exosomes promote bone formation, and that *in vivo* they increase the number of trabeculae and GHPA exosome-induced osteoblast proliferation *via* increased cell viability and DNA replication (54). Furthermore, they showed that exosomal hsa-miR-21-5p plays an important role in this process (which is distinct from that of the GH/IGF-1 axis), providing a novel mechanism for acromegaly development.

#### Potential treatment therapy for hormone-secreting PAs

Several groups have reported tumor-inhibiting ncRNAs and potential tumor treatment therapy in PAs (**Table 1**; **Figure 3D**).

### PRLPAs

Drug treatment is the first-line treatment therapy for PRLPA patients. However, some patients suffer from drug resistance. Exosome carrying tumor suppressors could provide a potential therapy for those patients. Wu et al. reported that H19 acts as a tumor suppressor in various tumors including PRLPAs (24, 60, 61). Importantly, lncRNA H19 levels in exosomes were dramatically correlated with the prognostic outcome of PRLPA patients. Furthermore, exosomal H19 and cabergoline treatment was shown to exhibit synergistic therapeutic effects (52). Mechanistically, H19 was shown to inhibit 4E-BP1 phosphorylation (52). In addition, a recent study by Zhao et al. reported that both miR-149 and miR-99a-3p present in exosomes showed suppressive effects on *in vitro* cell viability, metastasis, tube formation ability, and on *in vivo* tumor growth and angiogenesis (46). Mechanistically, exosome-driven miR-99a-3p inhibited PA cell growth *via* regulating the expression of *Nova1*, *Dtl*, and *Rab27b* (46). Based on these findings, since exosome is a promising delivery, we propose that those exosomal ncRNAs constitute suitable candidates as tumor inhibitors to treat patients with PRLPAs.

#### GHPAs

Pituitary adenoma is a common benign tumor. Interestingly, Zhou et al. reported that exosomes of GHPA cell line treated colon cancer cell line (HCT116 cell line) showed decreased malignancy *in vitro* and decreased tumor metastasis in HCT116 xenografted nude mice (11). This decreased malignancy occurs proposed through altered gene expression in the p53 and MAPK pathways (11). In addition, Zhao et al. indicates that low miR-423-5p levels promote tumorigenesis in somatotropic adenomas (53). Hence, exosomes containing inhibitory lncRNA or miRNAs disrupt PA malignancy. Based on these findings, exosomal ncRNAs constitute suitable candidates as tumor inhibitors to treat patients with GHPAs or some malignancy tumors.

## Conclusion

The studies of exosomal RNAs expand our understanding of the regulatory mechanisms in PAs. As exosome purification and detection technology developed in the past decade, it become easier to understand the precise contributions of exosomal RNAs in tumor genesis and development. In this review, we highlight the exosomal RNA functions as early diagnosis markers or invasive biomarkers in NFPAs. Since NFPAs are typically difficult to diagnose, this is an especially important finding. In addition, exosomal RNAs play distant regulation of bone formation in GHPAs. This propose novel insight which is different from traditional concept; Finally, tumor suppressor RNAs detected in exosome could be therapeutic applications. Since exosomes are promising vehicles for drug delivery, those RNAs detected in PA exosome propose important clinical application in the future. Although current investigations on exosome in PAs is limited, and it has not been fully translated into clinical applications. However, the current detections offer novel ideas and perspectives on the progress and treatment of PAs. The involved exosomal RNAs can also be exploited as reliable diagnostic biomarkers and therapeutic therapy in PA. Those investigations largely promote the use of exosome and their contents in both clinical diagnosis and treatment of this tumor.

## Author contributions

XB, RW, MF, and PL conceived of the project. MC wrote the manuscript. SJ, XG, PL, MF and JG modified the manuscript. All authors have read and approved the final version of manuscript. All authors contributed to the article and approved the submitted version.
